# Factors associated to the use of insecticide treated nets and intermittent preventive treatment for malaria control during pregnancy in Cameroon

**DOI:** 10.1186/s13690-016-0116-1

**Published:** 2016-02-01

**Authors:** Ngimuh Leonard, Fokam Bertrand Eric, Anchang-Kimbi K. Judith, Wanji Samuel

**Affiliations:** Department of Microbiology and Parasitology, University of Buea, Buea, 63 Cameroon; Department of Zoology and Animal Physiology, University of Buea, Buea, 63 Cameroon; Research Foundation in Tropical Diseases and Environment, P.O.Box 474, Buea, Cameroon

**Keywords:** Usage, ITN, IPT and pregnancy

## Abstract

**Background:**

Malaria in pregnancy has been shown to cause both maternal and infant morbidity and mortality especially in sub Saharan Africa. The World Health Organization therefore recommends the use of insecticide treated nets (ITNs), intermittent preventive treatment (IPT) and effective management of clinical malaria. The main aim of this study was to assess the coverage of ITN and IPT among pregnant women and the factors associated with their use in the Buea Health District of Cameroon.

**Methods:**

A cross sectional study was carried out from April to July 2014, in the Buea Health District which included 292 pregnant women attending antenatal care at clinics in the area. A structured questionnaire was use to obtain demographic data of participants and information on IPT and ITN use.

**Results:**

The Overall coverage rate of IPT was 88.7 % and 43.8 % for ITN while the overall non usage rate for IPT and ITN was 11.3 % and 17.5 % respectively. Occupation, educational level, trimester and number of ANC were statistically significant to ITN use by bivariate analyses while being a student/ unemployed (OR = 0.25, 95 % CI = 0.07–0.95)) was negatively associated to ITN use by multivariate analysis. For IPTp-SP, occupation of participants, educational level, trimester of pregnancy and number of ANC were statistically significantly by bivariate analyses while attending ANC just once (OR = 0.006, 95 % CI = 0.00–0.04) was negatively associated to IPTp-SP use by multivariate analyses.

**Conclusion:**

This study identified that the use of IPT was fairly good, while ITN use was still low despite their free distribution. Therefore, frequent antenatal care visits and involvement of participants in a potential income generating venture (Business or earning a salary) will increase IPT and ITN usage.

## Background

Malaria infection during pregnancy is a serious public health problem which can result in maternal and new born morbidity and mortality, especially in sub Saharan Africa where about 30 millions pregnant women are at risk of the disease yearly [1]. In Cameroon, just like in other endemic areas, malaria has been shown to be a cause of maternal anaemia, intra-uterine growth retardation, low birth weight, stillbirths and abortions [2, 3]. Pregnant women are more likely than non pregnant women to become infected with *Plasmodium falciparum* malaria and, once infected, there is a tendency toward increased severity of disease [4, 5]. The infection rate is higher among primigravidae than multidravid women [6].

The World Health Organization (WHO)’s recommendation for the control and prevention of malaria during pregnancy in areas of high to moderate malaria transmission in Africa is a package of intermittent preventive treatment (IPTp) with Sulfadoxine-Pyrimethamine (SP) and insecticide treated nets (ITNs) with effective management of clinical malaria and anaemia, which is commonly delivered through collaboration between malaria and reproductive-health programmes [5].

Studies show that the use of IPTp-SP for malaria in pregnancy in areas of high or seasonal transmission will result in lower placental infection rates, increase in both maternal haemoglobin levels and infant birth weight [7]. In addition, other studies in the mesoendemic area of the Thai-Burmese border and the Gambia [8, 9] found reductions in maternal anaemia and low birth weight with the use of ITNs.

So far, very little work has been done on the factors associated to the use of IPTp-SP and ITN during pregnancy in Cameroon. This study therefore seeks to assess the coverage rate and factors associated to the use of ITN and IPTp-SP for the control of malaria during pregnancy.

## Methods

### Study area

The study was carried out in the Buea Health District (BHD) that comprises of both rural and urban communities. It has seven health areas with a total of 21 recognised health facilities of which Six were purposively selected: Mount Mary Hospital, Buea Road HC, Regional Hospital Buea, Solidarity Health Foundation, Molyko HC, Mile 16 IHC, to have a spatial distribution of the health facilities.

### Study design and population

The study was a cross sectional hospital-based survey that contacted 410 pregnant women. However, 292 pregnant women met the inclusion criteria. That is being in their second or third trimester of pregnancy. There was no case of refusal. The survey was carried out from April to July 2014. The Cochran formula **(Z**^**2**^**x p (1-p)/d2 Where z = 1.96 for 95 % CI; p = ITN and IPT usage rate of 25 % and 90 % respectively; d = precision)** was used to estimate the minimum sample size assuming that, the proportion of pregnant women using ITN and IPT was 25 % and 90 % based on [10] and [11] respectively, with a 95 % confidence interval and a 5 % precision.

### Data collection

A closed ended structured questionnaire was designed and administered in English. However, for all illiterate women, questions were translated and the participants were interviewed in “Pidgin” (Creole). The questionnaire sought to obtain demographic data and data on IPTp-SP and ITN use. Before the study started the questionnaire was pre-tested out of the study site and upon analysis,,the results were promising and questions in which participants had difficulties understanding were rephrased before the final study. Hospital records of participants were used to confirm IPTp-SP use and the dosage. The Roll Back Malaria(RBM) partnership indicator for ITN use which is based on the proportion of pregnant women who slept under an ITN the previous night [12] was equally used to determine ITN use. The term ITN in this study was referred to nets that had been treated with insecticide and needed ongoing treatment. Or long lasting insecticide nets which are currently the most frequently distributed types of net in Africa [12]. The term free distribution net in this study refers to the door to door distribution as well as free nets received from the hospital. A sample size of 288 or 139 had been estimated to provide the desired outcome at 5 % precision, 95 % confidence level and an estimated usage rate for ITN of 25 % [10] and 90 % [11] for IPT.

### Ethical considerations

The study was approved by the Institutional Review Board of the Faculty of Health Sciences of the University of Buea. And an administrative authorisation was obtained from the Regional Delegation of Public Health, South West region and the District Medical Officer of Buea. Consent was sought from the different health facilities selected for the study. Informed consent was obtained from the women prior to their interview at the clinic.

### Data analysis

A template of the questionnaire was prepared using Epi Info version 3.4.3 statistical software and the data entered and subsequently exported to the Statistical Package for the Social Sciences SPSS version 20 (SPSS, Inc., Chicago, IL, USA) and analyzed. A descriptive statistical analysis was carried out on the use of ITN and IPT. Differences in proportions were analyzed using Chi square (*χ*^2^) tests. Multivariate analysis was done for all significant values obtained from bivariate analysis to get the best-fit model using unconditional multiple logistic regression. A *P*-value < 0.05 was considered a statistically significant association.

## Results

The majority of pregnant women in this survey were greater than 25 years, and the ages ranged from 18 to 48 years with a mean age of 26.73 ± 4.681. More than half of the women (60.6 %) were attending ANC for the third time or more; more than two thirds were enrolled or had attained secondary/high school or tertiary level of education, while 22.9 % had the primary level of education (Table 1). The overall use of ITN was 43.8 % and of IPTp-SP was 88.7 % while the overall non usage rate for IPT and ITN was 11.3 % and 17.5 % respectively. The use of both ITN and IPTp-SP was highest among multigravid women (42.3 %); women in their third trimester (53.6 %), women aged 26 years and above (72.2 %) and women who had three or more ANC attendance (67 %). The non usage of ITN and IPT was highest among women in their second trimester and first ANC attendance. A detailed comparison is found in table.

**Table 1:** Socio demographic characteristics of pregnant women attending ANC in the Buea Health District

Characteristics		Total enrolled	IPT users (%)	IPT Non users (%)	ITN users (%)	ITN Non users (%)
Total		292	259(88.7)	33(11.3)	128(43.8)	51(17.5)
Gravidity	Primigravid	97	94(36.3)	3(9.1)	27(21.1)	18(35.3)
	Secondigravid	92	76(29.3)	16(48.5)	44(34.4)	14(27.5)
	Multigravid	103	89(34.4)	14(42.4)	57(44.5)	19(37.3)
Age	<20	22	19(7.3)	3(9.1)	6(4.7)	3(5.9)
	21–25	98	92(35.5)	6(18.2)	29(22.7)	19(37.3)
	>25	172	148(57.1)	24(72.4)	93(72.6)	29(56.9)
Occupation	Students/unemployed	92	88(34)	4(12.1)	19(14.8)	26(51.0)
	Business	90	79(30.5)	11(33.3)	43(33.6)	12(23.5)
	Civil servants	58	50(19.3)	8(24.2)	34(26.6)	7(13.7)
	House wife/ farmers	52	42(16.2)	10(30.3)	32(25)	6(11.8)
Number of ANC Attendance	First	27	5(1.9)	22(66.7)	24(18.8)	1(2.0)
	Second	88	81(31.3)	7(21.2)	36(28.1)	12(23.5)
	Third or more	177	173(66.8)	4(12.1)	68(53.1)	38(74.5)
Education	Primary	68	56(21.6)	12(36.4)	36(28.1)	10(19.6)
	Secondary/high	126	108(41.7)	18(54.5)	62(48.4)	19(37.3)
	Tertiary	98	95(36.7)	3(9.1)	30(23.4)	22(43.1)
Trimester	Second	150	120(46.3)	30(90.9)	73(57)	19(37.3)
	Third	142	139(53.7)	3(9.1)	55(43)	32(62.7)

### Factors associated to ITN use

Bivariate analyses for factors associated with ITN use showed that the following factors were statistically significant at *P* < 0.05: occupation, trimester of pregnancy, source of net, number of ANC visits (Table 2).

**Table 2:** Factors associated to ITN use in the Control of malaria in pregnancy using Bivariate and Multivariate analysis

Factors		ITN use (%)	ITN Non users (%)	Significance	Multivariate analysis	
					OR (95 % CI)	*P*-value
Gravidity	Primigravid	27(21.1)	18(35.3)	χ2 = 3.92	NA	
	Secundigravid	44(34.4)	14(27.5)	P = 0.141	NA	
	muitigravid	57(44.5)	19(37.3)		NA	
Age	<20	6(4.7)	3(5.9)	χ2 = 3.92	NA	
	21–25	29(22.7)	19(37.3)	P = 0.141	NA	
	>25	93(72.6)	29(56.9)		NA	
Occupation	Students/unemployed	19(14.8)	26(51.0)	χ2 = 25.779	0.25(0.07–0.95)	0.042
	Business	43(33.6)	12(23.5)	P = 0.001	0.65(0.18–2.35)	0.511
	Civil servants	34(26.6)	7(13.7)		1.39(0.33–5.75)	0.654
	House wife/ farmers	32(25)	6(11.8)		REF	
Educational level	Primary	56(21.6)	10(19.6)	χ2 = 6.909	2.4(0.57–10.18)	0.234
	Secondary/high	108(41.7)	19(37.3)	P = 0.032	2.11(0.77–5.77)	0.147
	Tertiary	95(36.7)	22(43.1)		REF	
Trimester	Second	73(57)	19(37.3)	χ2 = 5.710	1.64(0.63–4.27)	0.312
	Third	55(43)	32(62.7)	P = 0.017	REF	
Source of ITN	Hospital	50(39.1)	18(41.9)	χ2 = 11.912	7.45(0.67–83.22)	0.103
	Free distribution	77(60.2)	20(46.5)	P = 0.003	8.06(0.75–87.04)	0.09
	Bought	1(0.8)	5(11.6)		REF	
Number of ANC visits	One	24(18.8)	1(2.0)	χ2 = 10.464	4.23(0.46–39.19)	0.204
	Twice	36(28.1)	12(23.5)	P = 0.005	1.46(0.53–4.01)	0.461
	Thrice or more	68(53.1)	38(74.5)		REF	

NA means not applicable and REF means reference group use for comparison

Again, using multivariate analyses to obtain the final best-fit model, only one factor (occupation of participants) was found to be independently associated with ITN use, with being a student/unemployed person negatively associated to IPT use.

### Factors associated to IPTp-SP use

Bivariate analyses for factors associated with IPTp-SP use showed that the following factors were statistically significant at *P* < 0.05: gravidity, occupation, educational level, trimester of pregnancy, number of ANC visits. Again, using multivariate analyses to obtain the final best-fit model, only one factor that is ANC attendance, was found to be independently associated with IPTp-SP use, with those visiting the clinics just once being negatively associated to IPTp-SP use (Table 3).

**Table 3:** Factors associated to IPTp use for malaria control in pregnancy using bivariate and multivariate analysis

Factors		IPT use (%)	IPT Non users (%)	Significant	Multivariate analysis	
					OR (95 % CI)	p-value
Gravidity	Primigravid	94(36.3)	3(9.1)	χ2 = 10.463	5.04(0.69-37.10)	0.112
	Secundigravid	76(29.3)	16(48.5)	P = 0.005	0.41(0.11-1.56)	0.190
	Multigravid	89(34.4)	14(42.4)		REF	
Age	<20	19(7.3)	3(9.1)	χ2 = 3.949	NA	
	21-25	92(35.5)	6(18.2)	P = 0.139	NA	
	>25	148(57.1)	24(72.4)		NA	
Occupation	Students/unemployed	88(34)	4(12.1)	χ2 = 8.135	1.64(0.28-9.67)	0.586
	Business	79(30.5)	11(33.3)	P = 0.043	4.31(0.85-21.86)	0.079
	Civil servants	50(19.3)	8(24.2)		0.51(0.07-3.92)	0.520
	House wife/farmers	42(16.2)	10(30.3)		REF	
Educational level	Primary	49(19.4)	12(36.4)	χ2 = 11.749	0.23(0.02-2.14)	0.195
	Secondary/high	108(42.9)	18(54.5)	P = 0.003	0.26(0.05-1.46)	0.126
	Tertiary	95(37.7)	3(9.1)		REF	
Trimester of pregnancy	Second	120(46.3)	30(90.9)	χ2 = 23.283	0.25(0.06-1.13)	0.071
	Third	139(53.7)	3(9.1)	P = 0.000	REF	
Number of ANC visits	One	5(1.9)	22(66.7)	Χ2 = 148.08	0.006(0.00-0.04)	0.001
	Twice	81(31.3)	7(21.2)	P = 0.000	0.39(0.10-1.56)	0.181
	Thrice or more	173(66.8)	4(12.1)		REF	

Where NA means not applicable and REF means reference group use for comparison

## Discussion

This study assessed factors affecting the uptake of IPTp-SP and use of ITN among pregnant women in the BHD. The overall usage rate obtained for ITN was 43.8 %, which is half of that reported in Sudan [13], but similar to that reported in Tanzania [14]. However, it was less than the target for the Abuja Declaration which was set at a target of 60 % ITN coverage. The usage rate of IPTp-SP was 88.7 % which is above the 80 % set by Roll Back Malaria Partnership for 2010, but less than the ambitious 100 % coverage set for 2015 by the same organization. The highest proportion of both ITN and IPTp-SP users were in the >25 year-old group,,women who were currently enrolled or had obtained the secondary or high school level of education that is multigravid women and those that had attended ANC three or more times. This might be because multigravid women already know the dangers of malaria in pregnancy. Also those with more ANC attendance had high chances of receiving more lectures on the importance of ITN and IPTp-SP and hence increasing their usage rate. Primigravid women were more likely to use IPTp-SP than ITN which is similar to findings made in a meta analysis [15].

The lone independent factor associated to ITPp-SP use was number of ANC attended, which is similar to findings made in Sudan [13]. This shows that the more the women go for ANC, the more knowledge they acquire and the more likely they are to accept and receive IPTp-SP. This therefore explains why those who attended ANC just once were negatively associated to IPTp-SP use. This finding is different from that reported in a study in Tanzania [16] where number of ANC attended had no effect on IPTp use.

Furthermore, occupation was the only independently associated factor to ITN use with being a student/unemployed negatively associated to ITN use.

Information or recall bias may have occurred during the study because the data on ITN use was based on interview.

No causal inferences may be drawn from these findings because of the weak nature of cross-sectional studies.

## Conclusion

This study identified that the use of IPT was fairly good while ITN use is still low despite their free distribution. However, the use of IPTp and ITN are independently affected by factors such as number of ANC attended and occupation of participant respectively. Effective public health education on the use of ITNs should be reinforced in addition to giving them out for free, to enhance accessibility to and use of IPT and ITN. It might be productive for public health authorities and actors to undertake mass awareness campaigns to educate mothers on the importance of regular ANC visits and IPT use targeting especially people with low levels of education, pregnant teenagers, students and the unemployed.
